# Incorporating Canopy Cover for Airborne-Derived Assessments of Forest Biomass in the Tropical Forests of Cambodia

**DOI:** 10.1371/journal.pone.0154307

**Published:** 2016-05-13

**Authors:** Minerva Singh, Damian Evans, David A. Coomes, Daniel A. Friess, Boun Suy Tan, Chan Samean Nin

**Affiliations:** 1 Forest Ecology and Conservation Group, David Attenborough Building, Department of Plant Sciences, Downing Street, University of Cambridge, Cambridge, CB2 3EA, United Kingdom; 2 École française d’Extrême-Orient, Siem Reap, Cambodia; 3 Department of Geography, National University of Singapore, 1 Arts Link, 117570 Singapore, Singapore; 4 APSARA National Authority, Angkor International Research and Documentation Centre, Siem Reap, Cambodia; 5 APSARA National Authority, Department of Forestry Management, Cultural Landscape and Environment, Siem Reap, Cambodia; Montana State University, UNITED STATES

## Abstract

This research examines the role of canopy cover in influencing above ground biomass (AGB) dynamics of an open canopied forest and evaluates the efficacy of individual-based and plot-scale height metrics in predicting AGB variation in the tropical forests of Angkor Thom, Cambodia. The AGB was modeled by including canopy cover from aerial imagery alongside with the two different canopy vertical height metrics derived from LiDAR; the plot average of maximum tree height (Max_CH) of individual trees, and the top of the canopy height (TCH). Two different statistical approaches, log-log ordinary least squares (OLS) and support vector regression (SVR), were used to model AGB variation in the study area. Ten different AGB models were developed using different combinations of airborne predictor variables. It was discovered that the inclusion of canopy cover estimates considerably improved the performance of AGB models for our study area. The most robust model was log-log OLS model comprising of canopy cover only (r = 0.87; RMSE = 42.8 Mg/ha). Other models that approximated field AGB closely included both Max_CH and canopy cover (r = 0.86, RMSE = 44.2 Mg/ha for SVR; and, r = 0.84, RMSE = 47.7 Mg/ha for log-log OLS). Hence, canopy cover should be included when modeling the AGB of open-canopied tropical forests.

## Introduction

Tropical forests sequestered 2.4 ± 0.4 pentagrams of carbon annually from 1990–2007, making them an important terrestrial carbon sink [1]. However, tropical land use changes, especially those leading to deforestation can contribute to carbon emissions from these carbon stocks [1]. From 2001–14, at 14.4%, Cambodia had the fastest acceleration in the annual forest rate per annum [2]. This potentially leaves behind a largely degraded landscape containing fragments of forest that have lower aboveground biomass (AGB) storage [3]. The development of methods to accurately quantify AGB is important in the context of carbon cycle studies and also for conservation policies that monetize the carbon held in the forests of developing tropical countries [4]. Traditional field based approaches for estimating AGB have provided the core data for forest carbon monitoring, but are limited their spatial scale, so are increasingly complemented by remote sensing data to generate maps of AGB [5].

Light detection and ranging (LiDAR) is a powerful tool for mapping AGB in tropical regions [6]. LiDAR-estimated top of the canopy height (TCH) has been found to relate closely to field-estimated AGB in some tropical forests [7]. LiDAR data have been extensively used for AGB monitoring of different types of tropical forests at different temporal and spatial scales. LiDAR data have been employed for mapping AGB across forests that have undergone varying levels of degradation in Indonesia [8] and across selectively logged forests of Brazil [9]. These data have also been employed for mapping variation in AGB at different spatial scales across different time periods in a closed canopy forest in French Guiana [10]. In addition to lowland tropical forest ecosystems, LiDAR derived metrics have produced robust AGB estimates for tropical sub-montane forests [11], open canopied forests [12] and tropical peat swamp forests [13].

Increasingly, LiDAR and aerial data are being used in combination with an image segmentation technique, Object Based Image Analysis (OBIA) to study forest structure variables and AGB in tropical forests [14]. A recent study used a combination of airborne LiDAR and aerial imagery for characterizing the horizontal structure of a tropical forest in the Comoros Islands using OBIA [15]. Canopy height and horizontal structure variables were employed for delineating the dominant vegetation classes in the study area and generating a vegetation structure based land cover map [15]. During the OBIA, image segmentation is achieved by defining individual, non-overlapping objects (tree crowns in this case) and extracting the relevant spatial and spectral attributes of these features [16]. OBIA predicted that the variables had a strong positive correlation with the field measured values of these attributes, in addition to producing tree crown polygons that closely resembled the real forest tree crowns [17]. However, OBIA based approaches have not been used extensively for the monitoring of forest biophysical parameters in the tropics, even though they can produce more robust AGB estimates, at least in temperate coniferous forests [18].

We hypothesize that horizontal canopy cover influences AGB stocks in human modified open-canopied forest ecosystems [19], in addition to TCH [7], which is a plot aggregate allometry only [20], LiDAR based tree height estimates scaled up from individual trees to plot scale will potentially provide more robust AGB estimates [21,22,23]. With this in mind, the research has made use of three predictor variables–percentage canopy cover, TCH, and Max_CH. The percentage canopy cover (derived from aerial imagery) is used to represent the horizontal structure of the canopy while the TCH represents the vertical structure of the canopy. Max_CH is a new metric that has been synthesized as a part of this research. This metric is based on maximum heights of individual trees derived by segmenting individual trees (from LiDAR using OBIA) and scaling up these maximum heights to the plot scale. Individual LiDAR tree heights have been employed for AGB estimation in the past [24].

The main aim of the research was to build an AGB estimation model for Angkor Thom using a combination of field to calibrate airborne LiDAR data. In particular, we highlight the role horizontal canopy cover plays in influencing AGB, alongside vertical canopy metrics. In addition to using the two different vertical canopy structure for AGB modeling, this study has compared field measured plot scale heights with the two LiDAR derived height metrics

## Materials and Methods

### Study area

**Fig 1:**
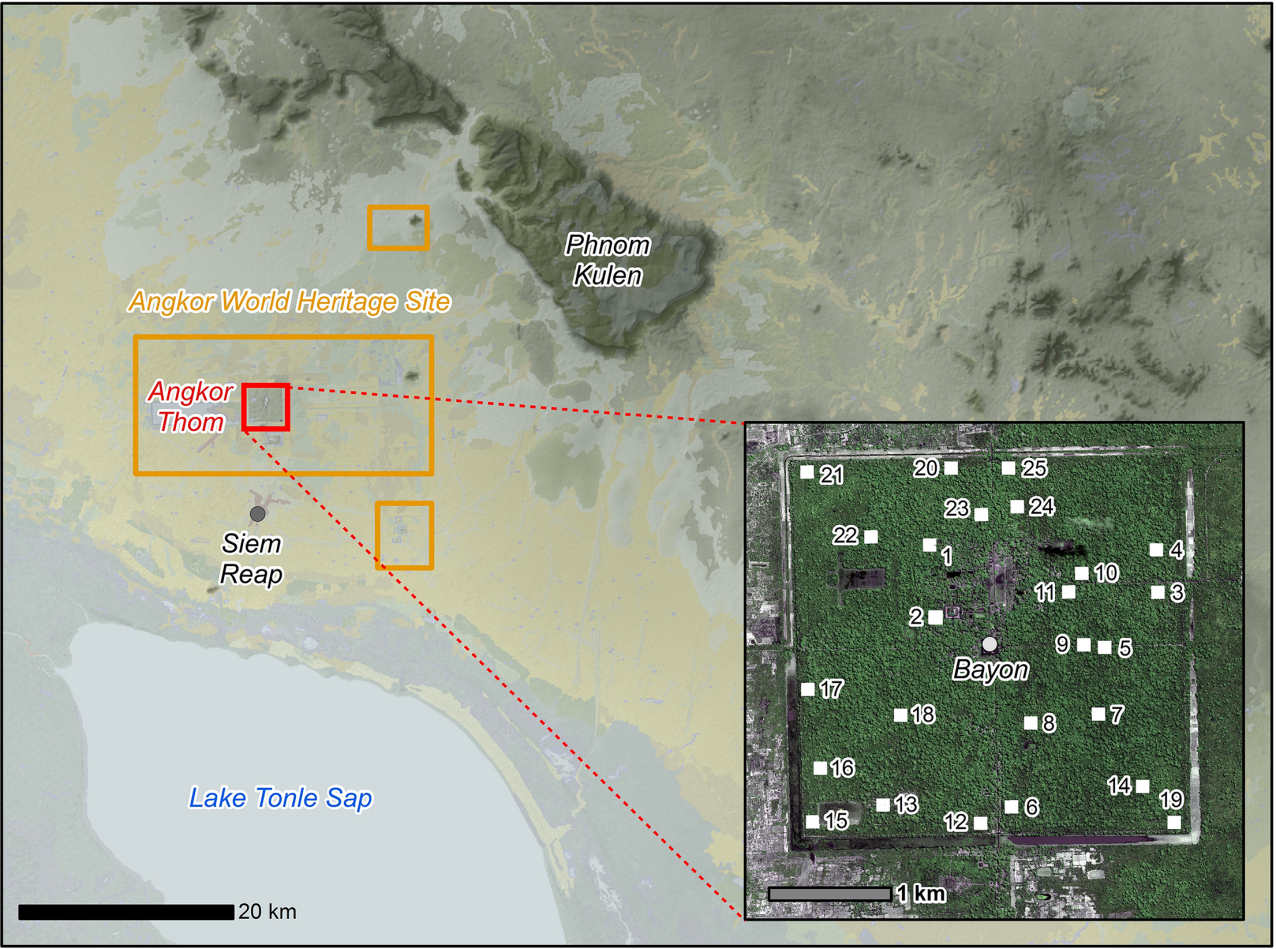
Map of the study area. Location of the Angkor World Heritage Site/ Angkor Archaeological Park, background data courtesy of NASA-SRTM/JICA-MPWT. Insert: Aerial view of the walled city of Angkor Thom showing the 25 one-hectare forest monitoring plots (IKONOS data courtesy of Space Imaging LLC).

The Angkor Thom complex within the World Heritage Site of Angkor (Siem Reap Province, Cambodia) covers 9 km^2^ (red box, Fig 1) and comprises of several important archaeological sites, including the iconic Bayon temple. Angkor Thom is an important heritage site, characterized as a “living landscape” within which management to conserve biological diversity must be reconciled with supporting cultural/religious beliefs and livelihoods [25]. Angkor Thom is one of 33 tropical sites, covering 26 million hectares of land that have been designated as World Heritage Sites (WHSs) since 1999. The forests of Angkor Thom and the wider Angkor Archaeological Park are moist tropical forests that experience strong seasonal variations in rainfall. These forests lie within the Central Indochina Dry Forest eco-region characterized by dry Dipterocarp forests and are vital components of this ecological province [26], but have come under pressure from increasing human population density, large-scale resource extraction, deforestation and degradation [27].

### Field data

Twenty five one-hectare plots were established randomly within the forested portion of Angkor Thom (see Fig 1). A one-hectare plot size was selected because the RMSE of LiDAR-derived AGB estimates decreases exponentially with increasing plot size, and previous work suggests that uncertainty reaches acceptable level at this area [28,29,6]. Stratification was conducted based on the four forest quarters created by the north, south, east and west roads of Angkor Thom. The forested areas leading towards Angkor Thom have faced different levels of anthropogenic disturbance owing to their varying distances from human settlements. Plots were randomly located within each of the quadrants. A preliminary survey of the forests indicated significant anthropogenic disturbance.

Plots were sampled in December 2013 using the procedures of Marthews et al. [30]. Stem diameter at breast height (DBH, in *cm*) was recorded for all trees where DBH ≥ 10 cm (see S1 Table). Total tree height (H, in *m*) of 150 randomly selected trees was measured using a clinometer [31], which can produce robust results even when the crown top is not visible [32], and log-log linear regression was used to derive the following relationship between DBH and H (Eq 1), as recommended by Feldpausch et al. [33], with R^2^ = 0.75, p<0.01:

ln(H)=0.292×ln(DBH)+2.1 (1)

This equation was then used to estimate the heights of all remaining trees in the plots, from their measured DBHs. The aboveground biomasses of all individual trees in the plot (AGB_tree_ in kg) were then calculated using the standard allometric equation (Eq 2) for moist forests of Chave et al. [34]:

$$AGB=0.0673\times \rho \times {\left({DBH}^{2}\times H\right)}^{0.976},$$ (2)

where, ρ is specific wood density; a mean value of 0.57 Mg/m^3^ was used as the value for individual tree species in the study area [35].

### Airborne imagery

**Fig 2:**
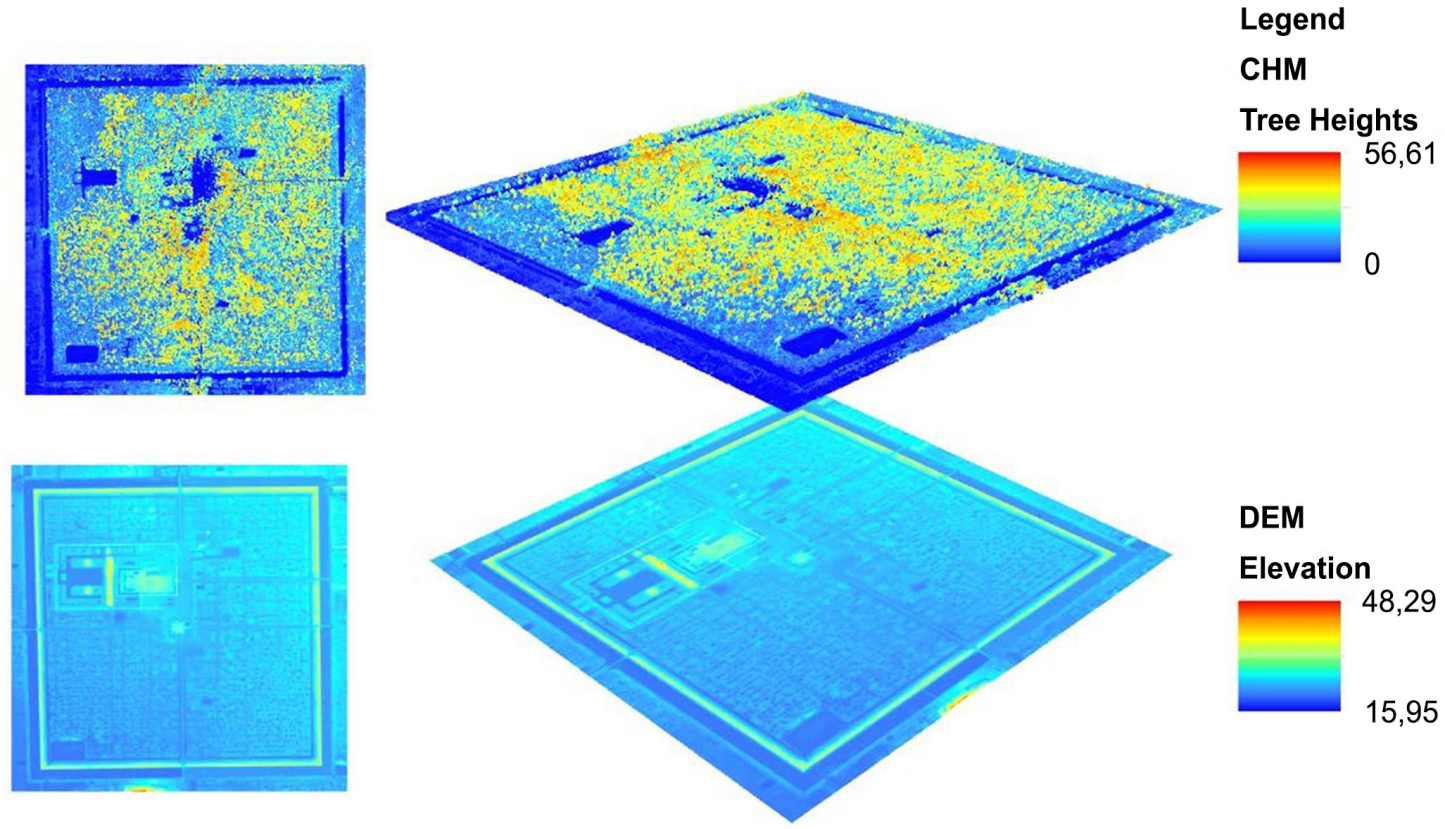
The Digital Elevation Model and the Canopy Height Model of the study area.

LiDAR data were acquired in April 2013, when there was the least amount of leaf cover on the trees, using a helicopter-mounted Leica ALS60 laser system. A Honeywell CUS6 inertial measurement unit was used to register aircraft orientation at 200 Hz [36], and absolute positional information was acquired using a Novatel L1/L2 GPS antenna. A flying height of 800 m above ground level and an average speed of 80 km/h were chosen, with a field of view of 45° for the laser scanner and 46° for the camera equipped with a 60-mm lens. The pulse rate of the ALS60 was 120 kHz, with full waveform acquired across a swath width averaging 650 m. The aircraft flew adjacent flight lines in opposing directions with a significant overlap between swaths, resulting in about four returns/pulses and a point density of about 12 points m^-2^. The LiDAR data were divided into ground and vegetation returns, using method [36]. Ground returns were used to derive a Digital Elevation Model (DEM) (Fig 2), while vegetation returns were used to generate a Canopy Height Model (CHM) giving the upper boundary of the canopy [37]. The CHM and DEM were generated using FUSION/LDV v.3.42 [38] at a resolution of 1m. TCH was obtained from the CHM clipped for each plot, as described by Mascaro et al. [7].

Object Based Image Analysis (OBIA) was used to delineate individual tree crown from the LiDAR data. The approach divides the image into non-overlapping regions or segments, extracts multiple pieces of information from each segment (e.g. shape, size, texture), and uses this information in conjunction with spatial context to categorize and collate individual segments into discrete objects [14]. Multi-resolution segmentation was conducted using eCognition software [14], where significant objects such as tree crowns were segmented one at a time, instead of attempting to segment the entire image simultaneously [17]. Individual tree crown-delineation of the entire LiDAR survey produced maximum tree heights of individual trees within each plot, which were averaged to give a plot-scale maximum canopy height estimate (henceforth referred to as Max_CH). This is more of an individual tree approach as compared to TCH which is a plot scale metric.

### Aerial Imagery

Very High Resolution (VHR) imagery was acquired concurrently with the LiDAR data using a 40-megapixel Leica RCD105 medium-format camera. The imagery comprised of three bands (red, green and blue), with a spatial resolution of 8cm. Radiometric correction and orthorectification were conducted by the data provider.

Manual digitization was employed to estimate percentage canopy cover from the VHR imagery, which is suitable for the delineation of individual trees as solid objects providing complete cover [39]. The summed area of all the digitized tree crowns within a plot, divided by the area of the plot, gives fractional canopy cover [40]. This method allows us to extract trees canopies accurately and leave aside bushes, shrubs (small vegetation) and shadows. The canopy cover extracted from the VHR imagery was obtained by taking 20–25 densitometer readings at random locations within each plot [39] (See S2 Table).

## Statistical Modeling

Multiple linear regression is the most common methods used to generate LiDAR-based biomass estimation equations. Arguably, however, relationships like these arise from fundamentally complex processes, with a number of interacting and intertwined drivers behind them. In contrast, machine learning methods are based on assumption that processes are unknown and likely to be complex. Support vector regression (SVR) is generally considered to produce robust results in the context of remote sensing. SVR deals with the inherent complexity and non-linearity of the data by using kernel functions to map the original input space into a new feature space with higher dimensions [41,42].

Previous work suggests the relationship between field-estimated AGB and LiDAR-estimated TCH is best described by a power law relationship [43]. In order to implement this, a log-log linear regression model was fitted, of the form [10]

ln(AGB)=a+bxLn(PredictorVariable)+e, (3)

where *a* and *b* are parameters and the residuals e are normally distributed and homogeneous. This relationship was extended by including additional variables such canopy cover and Max_CH in the linear model [10]. Alternative regression models were explored that included Max_CH instead of TCH; five log-log linear models were developed including different combinations of the three predictor variables. In all these cases, back-transformation was carried out using the Baskerville correction which produced the following model (Eq 4) [10] where RSE is the residual standard error.

$$AGB=exp(a+\frac{{RSE}^{2}}{2}+b\;*\;Ln(predictor\;variable))$$ (4)

In addition, SVR modeling was carried out (with radial bias function or RBF as the kernel) using the *caret package* of the R statistical framework [44]. In all, five SVR based models were developed which used different combinations of the three predictor variables under consideration. A total of ten models were compared—five utilizing OLS regression (log-log) and five utilizing SVR.

In order to assess the performance of the ten AGB models developed as a part of this research, leave one out cross validation (LOOCV) was implemented. The basic principle of this technique is that one observation is sequentially removed from the dataset and the model is fitted to the remaining data points. The model thus developed is implemented on the datum that was left out in order to produce a predicted value [45]. LOOCV was implemented in order to reduce overfitting and to provide unbiased error estimates [41]. The technique then averages the test error rate observed in each of the k iterations so as to get the cross-validation of the test error rate [46]. For implementing this technique in predictive regression models, regression is conducted with AGB values of the remaining plots (n-1) and the predicted AGB is obtained for the plot that was left out. This is conducted for all the data points [47]. Pearson correlation coefficient (between field and predicted AGB), Root Mean Square Error (RMSE), %RMSE, Mean Absolute Error (MAE), lower and upper RMSE and %bias were used to evaluate predictive AGB model performance. Models with relatively low MAEs, %RMSE, %bias and high Pearson’s correlation coefficient are considered preferable. Percent bias was evaluated by evaluating the percentage overestimation or underestimation of predicted AGB compared with field AGB values [48]. In order to determine the independent and joint contribution of the explanatory variables as predictors of field AGB, hierarchical partitioning analysis was implemented through the hier.part package of R [49]. Hier.part uses % RMSE as a goodness of fit measure and allows the calculation of both the independent and joint contributions of the predictor variables. The former quantifies the % contribution of independent variables in explaining variance while the latter quantifies the relative contribution of predictor variables. Additionally, Random Forest (RF) was also used for identifying the % contribution of individual variables

The research examined whether field measured tree height, Max_CH and TCH are significantly different using a Friedman test; a non-parametric test which is appropriate for paired data.

## Results

Field measured tree heights (19.53±0.2m), TCH (18.84±1.17m) and Max_CH (20.2 ± 1.03m) were not statistically different from each other (Friedman test, p>0.05). The mean height of trees delineated within the LiDAR imagery (i.e. Max_CH) and average field tree heights had a mean difference of 60 cm. The average difference between field-measured height and TCH was slightly greater, at 69 cm, which is to be expected because TCH was integrated over the entire canopy, not just the tree tops.

### Performance of the AGB estimation models

**Fig 3:**
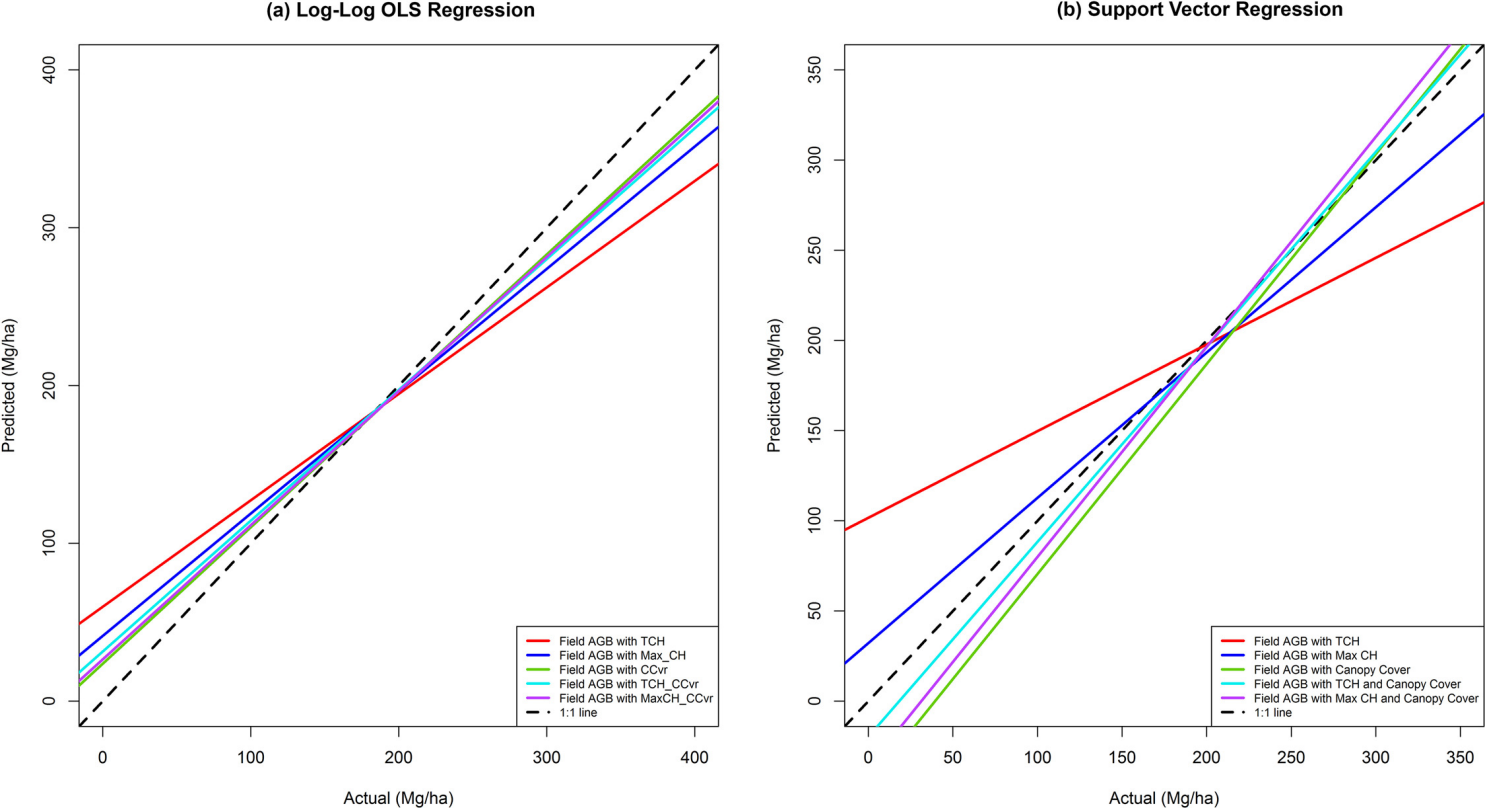
LiDAR predicted AGB estimates compared with the field estimates for the ten models.

Aboveground biomass (dry mass) ranged between 49–346 Mg/ha over the 25 plots (see S3 Table). Comparison of five least-square-regression models and five SVR models indicated that the best-supported model used log-log regression and had canopy cover as the only explanatory variable, closely followed by the SVR model that included both Max_CH and canopy cover (Table 1, Fig 3). The coefficient estimates, p values and R^2^ of the five log-log linear regression models are provided in S4 Table.

**Table 1:** Summary of statistical performance of the ten alternative models used to predict AGB from aerial remote sensing data. The statistics include Pearson’s correlation coefficient (r) between 25 observed and predicted AGB values, Root Mean Square Error (RMSE), % RMSE percent bias, MAE, lower and upper RMSE.

Model	*r*	RMSE	%RMSE	%Bias	MAE	RMSE lower	RMSE upper
**OLS Regression (log-log)**							
TCH	0.54	74.2	48.4	2.7	58.4	54.7	96.7
Max CH	0.66	65.1	37.0	1.6	48.7	48.6	81.3
Canopy Cover	0.87	42.8	33.8	1.6	31.8	31.0	55.5
TCH+Canopy Cover	0.84	48.4	35.8	1.1	35.9	34.7	62.9
Max CH+Canopy Cover	0.84	47.7	34.7	1.6	35.4	33.6	62.2
**SV Regression**							
TCH	0.23	84.5	85.3	-0.6	67.9	64.1	106.7
Max CH	0.40	77.8	83.6	3.6	67.4	59.9	95.9
Canopy Cover	0.82	50.7	51.4	6.2	39.8	39.7	62.3
TCH +Canopy Cover	0.82	48.2	50.9	1.9	38.2	36.1	62.2
Max CH+Canopy Cover	0.86	44.2	40.1	2.0	34.9	30.5	55.1

Fig 3 graphically compares the performance of the ten models, showing the regression lines for the field and LiDAR-predicted AGB using least squares regression (Fig 3A) and SVR (Fig 3B). The lines depict deviation of predicted AGB values from field AGB values. The lines were compared with the 1:1 relation (black dashed lines). From the ten models, the most parsimonious model was log-log OLS model comprised of canopy cover only. This model approximated the field AGB values with highest accuracy. After the log-log OLS canopy cover only model, the SVR regression and log-log OLS regression with Max_CH and canopy cover gave the closest prediction of field AGB values. Further, the predictive model with canopy cover only had a low value of percent bias (+1.6%), indicating small deviation from the expected AGB values. Variable importance analysis carried out using hier.part showed that in terms of % individual contribution canopy cover was more important than Max_CH and TCH (46.4, 24.8 and 13.1%, respectively) in explaining the variation in field AGB respectively. Canopy cover, Max_CH and TCH have a joint contribution of 29.7%, 29.8% and 21.6%. Random Forest (RF) % contribution of variable importance was 32.46%, 25.00% and 21.55% for aerial canopy cover, Max_CH and TCH respectively.

## Discussion

### AGB Modeling in Angkor Thom

The canopy cover–an index of horizontal variation in canopy structure–has the highest % independent contribution in explaining AGB variation. The best performing model was the log-log OLS model with canopy cover only. Additionally, the predictive models containing canopy cover have performed better than those with LiDAR height measures only. This research has established the importance of canopy cover for estimating AGB values in an open canopied forest. Recent works used combinations of vertical height and canopy cover metrics to improve AGB estimations such as in the works of Singh et al. [12] and Li et al. [50] and suggest the inclusion of horizontal canopy structure estimates (such as canopy cover) improves AGB prediction models for more open canopied ecosystems. These results may be explained on the basis of Drake et al. [19] according to which horizontal variables, such as canopy cover, play an important role in explaining AGB variation in open-canopied tropical forests. This maybe corroborated by research done by Coomes et al. which demonstrated that canopy cover and structure related variables (such as NDVI) are effective for modeling AGB in open shrub-lands [51]. The role of horizontal canopy structure in complementing LiDAR derived vertical structure for landscape scale forest structure has been previously reported [19,15,50]. Horizontal variables are important in explaining variation in AGB stocks and their exclusion from AGB estimation models can reduce accuracy [52]. Future AGB modeling in moist tropical ecosystems would benefit by using horizontal variables such as canopy cover either alone or in conjunction with vertical structure variables to produce robust AGB models.

While the difference between the five top performing models is not very high, they do present interesting insights which maybe be used to inform future research. For both categories (log-log regression and SVR), models with Max_CH produced slightly better results than Asner’s TCH metric. Scaling up from individual tree level to plot scale is a more intuitive approach as it closely follows the process of plot scale field AGB estimation. The latter also involves calculating the AGB of individual trees and scaling up to plot scale. Research by Colgan et al. [53] indicates that inclusion of OBIA derived maximum height metrics significantly reduces error in the AGB models as compared to an average height metric. Our research establishes the utility of individual tree height metrics in approximating average field tree heights and producing robust AGB predictive models. Upper layers of the canopy intercept most of the laser shots, hence it is expected individual LiDAR trees heights will correspond more strongly with the field measured tree heights and AGB estimates [24]. These results are in agreement with a similar study based in the temperate forests of Canada, which also utilized OBIA derived parameters from aerial and LiDAR data to develop AGB models [54]. The said model was developed using LiDAR data that covered 8.8% of the study area to produce a landscape wide estimate of AGB and volume in Canada [54]. In this research, Max_CH has been synthesized using pre-existing algorithms (OBIA in this case), for future research it would be informative to examine different ways of extracting individual tree characteristics, such as 3D segmentation directly from LiDAR point clouds and relating them with field data [17]. The top 5 AGB models predicted AGB values accurately with slight over estimation. However these values are well within range of AGB biases reported for other ecosystems [43].

It must be noted that the canopy metrics deployed here are not the only horizontal and vertical metrics that can be used for AGB modeling. Hansen et al. [11] discovered that variables representing lower parts of the canopy and canopy density predicted AGB density better than height metrics in a sub-montane tropical forest in Tanzania. Two vertical height metrics- first quartile heights and variance of heights above ground returns were used to model AGB variation in a selectively logged forest in Brazil [9]. Caution is needed when interpreting these results as more data are needed before being scaled in order to identify the environmental and biophysical factors which influence AGB stocks and which models are more appropriate for the wider ecological province of Central Indochina Dry Forests. For instance, while a canopy cover only model has produced robust AGB estimates for this ecosystem, it is important to test the ability of canopy cover based AGB models in predicting the AGB values of other forest types, including forest types with varying canopy covers and heights.

### Limitations of AGB modeling

There are several sources of uncertainty in remote sensing forest AGB monitoring studies. These include field measurement errors, plot location errors and errors stemming from the selection of allometric equations. These in turn can reduce the accuracy of the AGB estimates [55]. Many forest remote sensing studies suffer from the lack of differential GPS geo-located plot locations. The use of a handheld GPS (such as the one used in this study) can produce inaccurate plot locations in dense, close canopied forests [21]. However, in this work, the forest plots did not have a dense canopy cover and the plot locations were visually cross-checked with the locations of important monuments and other easily identifiable structure.

This study applied the general allometric equation recommended by Chave et al. [34]. However, AGB estimates are often hindered by a lack of site- and species-specific equations. Although local allometric models can produce more accurate AGB estimates, the development of such allometry needs substantial field data [56].

Both field measured and LiDAR predicted tree heights have associated measurement errors, which in turn can affect the efficiency of the AGB prediction model [6,57]. For instance, the precision of individual tree height measurements can vary between 3%-20% which in turn translates into 5%-9% uncertainty in AGB estimates at 1ha scale [58]. This study has used clinometers for measuring tree heights in the field. A recent comparison of ground-based methods found that clinometer based- trigonometric methods resulted in no systematic bias (non-laser method), or resulted in a small underestimate of actual tree height (ground laser-based methods) compared to heights measured from an observational tower [32]. Clinometer measured tree heights essentially produces 1:1 correspondence with actual tree heights, but the height of taller trees can significantly be overestimated. This approach produces low systematic error but high random errors [32]. Including height (whether field measured or DBH-height equation derived) can significantly reduce uncertainty in field AGB measures [33]. These meta-scale findings are further corroborated by Rutishauser et al. who argue that while measuring tree heights in tropical ecosystems (such as Indonesia) is fraught with uncertainties, including them reduced uncertainty in the overall AGB model [59]. This certainly builds a case for developing site specific H-DBH relationships. In this research at a plot scale, field measured tree heights, Max_CH and TCH did not vary significantly. However, the impact of high random errors produced as a result of taller trees needs to be evaluated in detail, as forests of SE Asia on an average have trees taller than those found in other tropical regions [33].

### Comparison to other LiDAR-based studies of tropical forest AGB and structural dynamics

The present study area encompasses a wide range of AGB values. In spite of a ban on logging, illegal resource extraction persists in many parts of the study area which explains such a wide variation in AGB values. Previous findings in Borneo indicate that selective logging can lead to 55%–66% AGB loss [60]. The wide range of AGB values in the study area is similar to the range of AGB values for mixed evergreen and semi-evergreen forests identified in the national-scale assessments of AGB in Cambodia [61]. This study confirms that local spatial variation in forest cover and AGB is fraught with uncertainty, which in turn has implications for carbon conservation activities such as REDD+ [62]. Such wide variation of AGB in a relatively small area indicates the need to build high resolution AGB maps at a local scale (based on locally relevant predictor variables) and scaling these to landscape or regional scale.

The mean AGB of the semi-evergreen forests of Angkor Thom was 194.53 Mg/ha, or 97.265 Mg C/ha (above ground carbon stocks are half of total AGB). This value of above ground carbon stocks is comparable to the carbon stock values reported for other sites in continental and insular SE Asia [63,64]. It also showed that the airborne data derived variables had a strong strength of association with field measured AGB [13]. Furthermore, both AGB variation and the effect of spatial patterns of degradation on AGB variation were quantified to a high level of accuracy using LiDAR data collected over lowland and peat-swamp forests in Indonesia [8]. On the basis of the research presented in this paper and previous studies, airborne data such as LiDAR are appropriate in examining forest structure, AGB variation and degradation patterns in tropical forest ecosystems, including those in Cambodia.

## Conclusions

This study develops an AGB-estimation method for the temple forests around Angkor Thom. Two distinct approaches are used for AGB modeling—log-log regression and the support vector regression. The canopy cover-only AGB model and the models that included of canopy cover along with height produced robust estimates AGB in the open canopied tropical forests of Angkor Thom compared to height only models. Inclusion of the two height metrics (the plot scale TCH and individual tree height based Max_CH) in the different models confirm the role of the latter, an individual scale metric scaled up to plot level in predicting AGB variation with a higher level of accuracy compared to the plot scale TCH. It is expected that scaling these models will produce improved AGB estimates at landscape scales for ecosystems that suffer from high levels of anthropogenic disturbance. This study has provided valuable insights into the structural variation present in the said ecosystem and the ability of airborne data to predict this. Conservation instruments like the REDD+ require highly accurate quantification of parameters such as AGB and carbon, which can be obtained by using a combination of field and airborne data. Further works will extend airborne data-based approaches to open-canopied tropical forests

## Supporting Information

S1 Table Measured DBH and Tree Heights. (DOCX)

S2 Table Ground canopy cover and aerial canopy cover for each of the 25 plots. (DOCX)

S3 Table Field height measurements, top of the canopy height (TCH), maximum canopy height (Max_CH) measurements and field measured above ground biomass for each of the 25 plots. (DOCX)

S4 Table Coefficient Parameter Estimates and R^2^ of Log-Log Linear Regression models. (DOCX)
