# Supplementary material for: Incorporating Canopy Cover for Airborne-Derived Assessments of Forest Biomass in the Tropical Forests of Cambodia
Source: PLoS One. 2016 May 13;11(5):e0154307. doi: 10.1371/journal.pone.0154307 (PMC4866690; doi:10.1371/journal.pone.0154307)
Supplement: S1 Table — (DOCX) [file pone.0154307.s001.docx]

**DBH- Height Relationship**

Tree heights of all the trees in the 25 plots could not be measured. Instead tree heights and DBH were measured for 151 trees. Size-stratified random sampling was carried out with the view of ensuring that both large trees as well as relatively smaller trees were included. See S1 Table.

**S1 Table. Measured DBH and Tree Heights**

| **DBH (in cm)** | **Tree Height (in m)** |
| --- | --- |
| 138.95 | 35.00 |
| 33.70 | 22.50 |
| 114.17 | 30.50 |
| 173.90 | 32.80 |
| 46.66 | 24.50 |
| 21.35 | 18.20 |
| 47.58 | 23.50 |
| 962.53 | 45.80 |
| 35.65 | 20.90 |
| 24.07 | 17.60 |
| 25.46 | 22.30 |
| 36.15 | 21.60 |
| 28.08 | 19.30 |
| 35.65 | 21.50 |
| 15.80 | 25.00 |
| 33.70 | 34.40 |
| 32.31 | 28.50 |
| 12.80 | 35.90 |
| 46.53 | 23.40 |
| 119.08 | 38.90 |
| 122.47 | 30.30 |
| 153.28 | 31.90 |
| 27.62 | 19.70 |
| 181.43 | 33.45 |
| 163.16 | 37.80 |
| 23.08 | 18.40 |
| 33.70 | 21.10 |
| 1420.50 | 49.10 |
| 251.55 | 35.43 |
| 56.51 | 24.79 |
| 32.25 | 22.30 |
| 164.41 | 32.40 |
| 24.67 | 19.80 |
| 132.66 | 30.87 |
| 58.13 | 24.99 |
| 108.42 | 29.50 |
| 38.96 | 24.60 |
| 121.34 | 30.23 |
| 27.51 | 22.30 |
| 173.12 | 37.90 |
| 44.50 | 39.43 |
| 39.95 | 33.40 |
| 144.51 | 38.80 |
| 35.70 | 37.55 |
| 43.38 | 22.90 |
| 118.90 | 46.50 |
| 9.07 | 11.74 |
| 52.79 | 24.30 |
| 120.76 | 30.20 |
| 28.48 | 19.90 |
| 47.80 | 57.80 |
| 108.90 | 33.35 |
| 14.73 | 15.20 |
| 125.03 | 30.45 |
| 54.69 | 24.55 |
| 173.94 | 32.80 |
| 125.03 | 30.45 |
| 54.69 | 24.55 |
| 107.30 | 45.00 |
| 31.76 | 20.68 |
| 46.11 | 23.34 |
| 124.53 | 30.42 |
| 124.33 | 30.41 |
| 67.31 | 26.03 |
| 294.37 | 36.55 |
| 4.25 | 6.33 |
| 3.46 | 4.88 |
| 5.86 | 8.63 |
| 57.09 | 24.86 |
| 88.85 | 28.01 |
| 98.00 | 30.42 |
| 142.00 | 47.88 |
| 29.00 | 26.03 |
| 16.00 | 20.25 |
| 78.00 | 29.39 |
| 162.00 | 35.30 |
| 123.00 | 33.07 |
| 8.00 | 14.15 |
| 11.50 | 16.89 |
| 78.10 | 30.32 |
| 13.00 | 18.05 |
| 16.10 | 19.30 |
| 14.10 | 19.40 |
| 30.00 | 22.90 |
| 44.00 | 26.82 |
| 20.00 | 20.85 |
| 14.00 | 17.40 |
| 27.80 | 23.97 |
| 80.10 | 30.15 |
| 20.00 | 19.90 |
| 8.00 | 13.92 |
| 10.00 | 15.27 |
| 27.00 | 22.41 |
| 22.10 | 20.96 |
| 7.00 | 14.55 |
| 37.10 | 24.69 |
| 13.00 | 18.47 |
| 11.00 | 16.77 |
| 16.50 | 20.07 |
| 26.10 | 23.29 |
| 127.00 | 33.79 |
| 11.50 | 16.23 |
| 23.10 | 22.12 |
| 28.00 | 23.57 |
| 11.00 | 17.59 |
| 14.00 | 19.31 |
| 11.00 | 17.34 |
| 9.00 | 16.29 |
| 19.00 | 19.70 |
| 10.00 | 16.11 |
| 10.00 | 16.04 |
| 17.10 | 19.38 |
| 11.00 | 16.43 |
| 11.00 | 15.74 |
| 12.00 | 18.14 |
| 13.10 | 18.16 |
| 11.00 | 16.57 |
| 18.00 | 19.16 |
| 16.10 | 19.74 |
| 22.00 | 20.89 |
| 10.00 | 16.24 |
| 13.10 | 18.18 |
| 23.00 | 21.51 |
| 10.50 | 16.46 |
| 9.00 | 14.45 |
| 13.10 | 18.51 |
| 10.00 | 15.04 |
| 12.00 | 17.70 |
| 12.00 | 18.11 |
| 140.00 | 33.35 |
| 8.00 | 14.02 |
| 8.50 | 14.26 |
| 15.00 | 17.84 |
| 64.00 | 28.70 |
| 10.00 | 15.45 |
| 11.00 | 17.34 |
| 11.00 | 16.57 |
| 12.10 | 18.00 |
| 14.00 | 19.34 |
| 42.00 | 25.98 |
| 28.00 | 23.88 |
| 24.00 | 21.96 |
| 24.00 | 21.96 |
| 18.10 | 21.00 |
| 21.00 | 20.89 |
| 10.00 | 15.41 |
| 9.00 | 14.61 |
| 14.00 | 18.37 |
| 12.10 | 17.33 |
| 19.00 | 20.50 |
| 14.00 | 18.41 |

As Feldpausch et al. recommends, regression relationship was derived between field measured tree height and natural log DBH in cm [63].

| $Ln(Height)=0.29\times Ln\left( DBH \right)+2.1$ |  |
| --- | --- |

(Here R^2^=0.75 and p < 0.001)

For trees whose actual heights had not been measured in the field, their heights were estimated using the DBH-Height relationship derived using the above equation.
