# Supplementary material for: Incorporating Canopy Cover for Airborne-Derived Assessments of Forest Biomass in the Tropical Forests of Cambodia
Source: PLoS One. 2016 May 13;11(5):e0154307. doi: 10.1371/journal.pone.0154307 (PMC4866690; doi:10.1371/journal.pone.0154307)
Supplement: S2 Table — (DOCX) [file pone.0154307.s002.docx]

Ground canopy cover and aerial canopy cover for each of the 25 plots. GRS Densitometer was used for estimating ground % canopy cover while the aerial % canopy cover was estimated using VHR imagery.

**S2 Table. Ground canopy cover and aerial canopy cover for each of the 25 plots.**

| **Plot** | **Ground Canopy Cover**  **(%)** | **Aerial Canopy Cover**  **(%)** |
| --- | --- | --- |
| 1 | 54.0 | 51.98 |
| 2 | 52.0 | 52.28 |
| 3 | 63.0 | 56.77 |
| 4 | 60.0 | 64.31 |
| 5 | 58.0 | 58.73 |
| 6 | 69.0 | 67.7 |
| 7 | 61.0 | 56.72 |
| 8 | 64.0 | 63.17 |
| 9 | 61.0 | 63.26 |
| 10 | 64.0 | 60.66 |
| 11 | 60.5 | 62.22 |
| 12 | 48.0 | 46.03 |
| 13 | 41.0 | 37.72 |
| 14 | 44.0 | 42.09 |
| 15 | 35.0 | 37.15 |
| 16 | 45.0 | 39.72 |
| 17 | 55.0 | 52.07 |
| 18 | 56.0 | 49.38 |
| 19 | 52.0 | 47.98 |
| 20 | 45.0 | 44.94 |
| 21 | 61.0 | 44.88 |
| 22 | 66.0 | 61.66 |
| 23 | 74.0 | 60.06 |
| 24 | 51.0 | 58.41 |
| 25 | 48.0 | 52.23 |
| **Mean (SE)** | **55.5 (± 1.88)** | **53.3 (± 1.78)** |
