# Supplementary material for: Incorporating Canopy Cover for Airborne-Derived Assessments of Forest Biomass in the Tropical Forests of Cambodia
Source: PLoS One. 2016 May 13;11(5):e0154307. doi: 10.1371/journal.pone.0154307 (PMC4866690; doi:10.1371/journal.pone.0154307)
Supplement: S3 Table — (DOCX) [file pone.0154307.s003.docx]

TCH was obtained from plot scale CHM as described by Mascaro et al.. Based on the OBIA analysis of the LiDAR data, the maximum tree heights of individual trees for each plot were averaged to give a plot-scale OBIA-based maximum canopy height estimate referred to as Max_CH. According to Asner et al. [60], the relationship between field AGB and TCH can be described by a power law relationship; AGB= a* TCH^b^. Both TCH and Max_CH were substituted into the stated power law. Analysis indicate that LiDAR derived height metrics (both TCH and Max_CH) have a moderate to strong positive correlation with field measured AGB. For AGB-Max_CH, Pearson’s correlation is 0.73 and for AGB-TCH, it is 0.61. Analysis also indicate that LiDAR derived height metrics (both TCH and Max_CH) have a strong positive correlation with aerial canopy cover. For aerial canopy cover-Max_CH, Pearson’s correlation is 0.82 and for aerial canopy cover-TCH, it is 0.74.

**S3 Table. Field height measurements, top of the canopy height (TCH), maximum canopy height (Max_CH) measurements and field measured above ground biomass for each of the 25 plots.**

| **Plot** | **Field Height (m)** | **TCH (m)** | **Max_CH (m)** | **Field measured**  **above ground biomass**  **(Mg/ha)** |
| --- | --- | --- | --- | --- |
| 1 | 20.52662 | 17.78 | 19.43 | 212.4821 |
| 2 | 21.60148 | 18.75 | 21.14 | 210.7716 |
| 3 | 19.7776 | 25.23 | 23.96 | 235.6992 |
| 4 | 19.29062 | 27.95 | 26.66 | 282.3808 |
| 5 | 20.62479 | 23.12 | 22.50 | 252.7813 |
| 6 | 21.12804 | 25.87 | 26.38 | 300.7502 |
| 7 | 21.11652 | 14.99 | 16.42 | 213.418 |
| 8 | 19.84588 | 20.02 | 19.13 | 290.0196 |
| 9 | 21.02919 | 26.44 | 27.32 | 233.4694 |
| 10 | 20.0263 | 10.00 | 26.19 | 274.7001 |
| 11 | 19.6669 | 22.30 | 23.25 | 275.8988 |
| 12 | 19.88214 | 20.48 | 21.24 | 114.6951 |
| 13 | 18.7779 | 11.77 | 13.72 | 92.12438 |
| 14 | 18.78856 | 16.06 | 16.63 | 93.83465 |
| 15 | 18.74431 | 9.13 | 9.76 | 58.78676 |
| 16 | 19.05711 | 9.99 | 10.96 | 67.11023 |
| 17 | 18.97239 | 20.25 | 23.16 | 206.8166 |
| 18 | 18.34453 | 21.59 | 22.62 | 144.0199 |
| 19 | 18.2671 | 15.72 | 17.15 | 144.7681 |
| 20 | 18.54816 | 15.39 | 16.87 | 133.8205 |
| 21 | 18.43745 | 10.32 | 11.28 | 49.45328 |
| 22 | 19.98666 | 27.77 | 26.26 | 204.4527 |
| 23 | 18.71568 | 22.01 | 21.22 | 292.4301 |
| 24 | 18.70956 | 19.29 | 19.97 | 346.3915 |
| 25 | 18.41973 | 18.75 | 20.00 | 132.0672 |
| **Mean (SE)** | **19.53 (± 0.2)** | **18.84 (± 1.16)** | **20.13 (± 1.01)** | **194.53 (± 17.17)** |

Mascaro J, Detto M, Asner G, Muller-Landau H. A Tale of Two “Forests”: Random Forest Machine Learning Aids Tropical Forest Carbon Mapping. PLOS one. 2014; 9(1): e85993.
