# Supplementary material for: Incorporating Canopy Cover for Airborne-Derived Assessments of Forest Biomass in the Tropical Forests of Cambodia
Source: PLoS One. 2016 May 13;11(5):e0154307. doi: 10.1371/journal.pone.0154307 (PMC4866690; doi:10.1371/journal.pone.0154307)
Supplement: S4 Table — (DOCX) [file pone.0154307.s004.docx]

S4 Table: Coefficient Parameter Estimates and R^2^ of Log-Log Linear Regression models

| **Model** | **a** | **B** | **c** | **Correction Factor** | **R^2^** |
| --- | --- | --- | --- | --- | --- |
| Log-log: TCH | 1.860 | 1.1387 | NA | 0.0830 | 0.487 |
| Log-log: Max CH | 0.464 | 1.5765 | NA | 0.0500 | 0.677 |
| Log-log: Canopy Cover | -6.050 | 2.8281 | NA | 0.0300 | 0.810 |
| Log-log: TCH/Canopy Cover | -5.600 | 0.1615 | 2.60 | 0.0307 | 0.815 |
| Log-log: Max CH/Canopy Cover | -4.911 | 0.4765 | 2.18 | 0.0282 | 0.829 |

In all the cases, the p-values were <0.01. Log-log linear model with with Max_CH and canopy cover was the best performing model out of the five linear regression models.
